# Variations of Major Product Derived from Conversion of 5-Hydroxymethylfurfural over a Modified MOFs-Derived Carbon Material in Response to Reaction Conditions

**DOI:** 10.3390/nano8070492

**Published:** 2018-07-05

**Authors:** Zhenhua Wang, Qianwang Chen

**Affiliations:** 1 School of Metallurgy and Materials Engineering, Chongqing University of Science and Technology, Chongqing 401331, China; 2 Chongqing Key Laboratory of Nano/Micro Composite Materials and Devices, Chongqing 401331, China; 3 Hefei National Laboratory for Physical Science at Microscale and Department of Materials Science & Engineering, Collaborative Innovation Center of Suzhou Nano Science and Technology, University of Science and Technology of China, Hefei 230026, China; cqw@ustc.edu.cn; 4 High Magnetic Field Laboratory, Chinese Academy of Sciences, Hefei 230031, Chin

**Keywords:** biomass, MOFs precursor, 5-hydroxymethylfurfural, catalysis, carbon material

## Abstract

In recent years, the conversion of 5-hydroxymethylfurfural (HMF) into 5-ethoxymethylfurfural (EMF) and ethyl levulinate (EL) has become an attractive biomass transformation route due to their potential applications in the energy and chemical industries. In this study, we have developed an effective method to prepare a catalyst for this reaction. Sulfonic-acid-functionalized carbon nanomaterials (C-SO_3_H), prepared from the direct pyrolysis of Metal-Organic Frameworks (MOFs) precursor Cu-benzene-1,3,5-tricarboxylate (Cu-BTC) followed by acidification with sulfuric acid, show excellent catalytic activity with a total yield higher than 90%. It is interesting that, different from the previous catalysts, a different major product—EMF or EL—can be selectively obtained by controlling the reaction temperature and time.

## 1. Introduction

For a long time, fossil energy shortage and environmental pollution have both been major problems besetting mankind. Much effort has been devoted to seeking abundant, renewable, and clean energy which can gradually replace fossil resources. As a widely sustainable and available carbon source, biomass is regarded as a potential alternative to fossil energy for the production of chemicals and liquid fuels [1,2,3,4,5,6]. In last several years, various chemicals have been successfully synthesized from biomass. Among them, 5-hydroxymethylfurfural (HMF) derived from biomass carbohydrate has been recognized as a significant platform compound. Due to its special structure with two functional groups combined with a furan ring, HMF can be transformed into a number of compounds with high potential applications in fuel or polymers, including γ-valerolactone, levulinic acid, ethyl levulinate (EL), 2,5-dimethylfuran (DMF), 5-ethoxymethylfurfural (EMF), and so on [7,8,9,10,11,12].

**Scheme 1:**
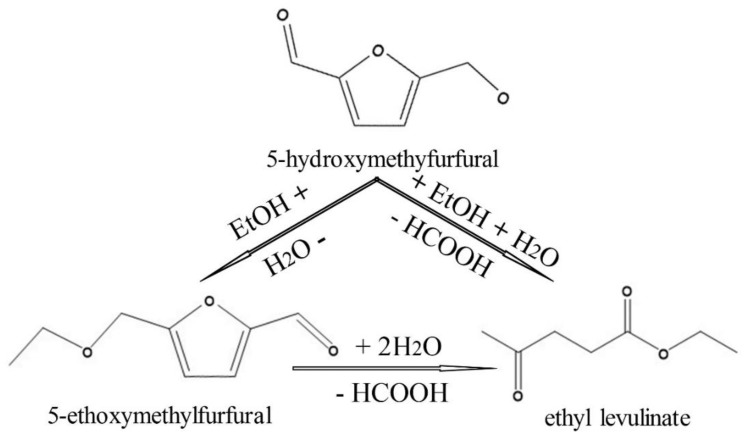
Conversion of 5-hydroxymethylfurfural (HMF) into 5-ethoxymethyfurfural (EMF) and ethyl levulinate (EL) in ethanol.

5-ethoxymethyfurfural (EMF), the etherification product of HMF, has attracted a lot of attention because of its potential applications in liquid fuel. EMF is present as a liquid with good oxidation stability, a high boiling point of 235 °C, and high cetane value, which makes it a good potential fuel and fuel additive. The energy density of EMF is 30.3 MJ/L, close to that of diesel (33.6 MJ/L), and significantly higher than that of ethanol (23.5 MJ/L) [13]. Meanwhile, the high oxidation stability of EMF can reduce the emission of soot, NO*_x_*, and SO*_x_* [14]. These features make EMF a potential fuel which can solve the problem of energy shortage and environmental pollution at the same time. In addition to EMF, ethyl levulinate (EL), as a diluent for biodiesel fuels with high saturated fatty acid content, was investigated as a novel, bio-based cold flow improver for use in biodiesel fuels [15]. Meanwhile, EL is used as an intermediate in the synthesis of more complex commercial products and spices in food additives [16]. EMF is generally generated by the etherification of HMF, while EL is produced via the reaction of HMF with ethanol or further decomposition of EMF as shown in Scheme 1 [17]. Therefore, EMF is usually produced along with EL.

In recent years, various acidic catalysts have been developed for the synthesis of EMF and EL from both HMF and fructose in ethanol. However, most of these catalysts have a common point: only one major product—EMF or EL—can be obtained. For example, catalysts such as Z-SBA-15 [11], Zr(O)Cl_2_/CrCl_3_ [18], ionic liquids (imidazolium propane sulfonic acids) [19], and heteropolyacid [20] can only selectively change HMF into EMF. On the other hand, the major product EL can be produced when sulfonic-acid-functionalized ionic liquids [21], CNT-PSSA [22], or SO_4_^2−^/ZrO_2_ [23] are used as the catalyst. As far as we know, different major products obtained from one catalyst in response to changing reaction conditions have been rarely reported yet. It will be a great prospect for industrial applications if the different products can be obtained by changing reaction conditions.

In this work, we found an interesting phenomenon. Sulfo-group-modified porous carbon nanomaterial (C-SO_3_H) was synthesized through the Metal-Organic Frameworks (MOFs) precursor, which exhibits excellent catalytic activity in the conversion of HMF into EMF and EL. Further, a different major product—EMF or EL—can be obtained by changing the reaction conditions.

## 2. Experimental Section

### 2.1. Preparation of the Catalyst

All chemicals were commercially available and used without any purification. In a typical synthesis of octahedral Cu-benzene-1,3,5-tricarboxylate (Cu-BTC) precursor, 1.82 g of cupric nitrate was dissolved in 50 mL methanol and stirred in ambient conditions for 20 min. After that, 0.875 g of 1,3,5-benzenetricarboxylic acid completely dissolved in 50 mL methanol was poured into the above solution under agitated stirring to form a blue turbid solution. The solution was aged at room temperature without any interruption for 12 h. The resulting blue precipitate was centrifuged and washed several times with methanol before drying in an oven at 60 °C. The precursor was annealed under an N_2_ atmosphere at 600 °C for 4 h using a heating rate of 5 °C/min. Then, the resulting sample was treated with sulfuric acid at 80 °C for 24 h to produce the sulfo-modified C-SO_3_H catalyst.

### 2.2. Characterization of the Catalyst

Powder X-ray diffraction (XRD) patterns were collected on a Japan Rigaku D/MAX-cA X-ray diffractometer (Tokyo, Japan) using Cu Kα radiation over the 2θ range of 10–70°, with working voltage of 40 kV and current of 200 mA. Scanning electron microscopy (SEM) images were taken using a JEOL JSM-6700M scanning electron microscope (Tokyo, Japan). X-ray photoelectron spectroscopy (XPS) was performed on an ESCALAB 250 X-ray photoelectron spectrometer (Waltham, MA, USA) using Al Kα radiation. Fourier transform infrared spectroscopy (FT-IR) spectrum Nicolet-8700 was determined using a Magna-IR 750 spectrometer (Waltham, MA, USA).

### 2.3. HMF Conversion into EMF and EL

In a typical experiment for the synthesis of EMF and EL from HMF, 0.5 mmol of HMF was dissolved in 4 mL of ethanol, and 40 mg of catalyst was subsequently added. The mixture solution was transferred into a Teflon lined autoclave with a capacity of 10 mL and heated at the reaction temperature for a period of time. After the reaction, the catalyst was removed by centrifugation and the yellow supernatant liquid was analyzed by Trace GC/ISQ MS. The yield of EMF and EL was quantified by GC-9860 from the calibration curve with dodecane as the internal standard.

## 3. Results and Discussion

**Figure 1:**
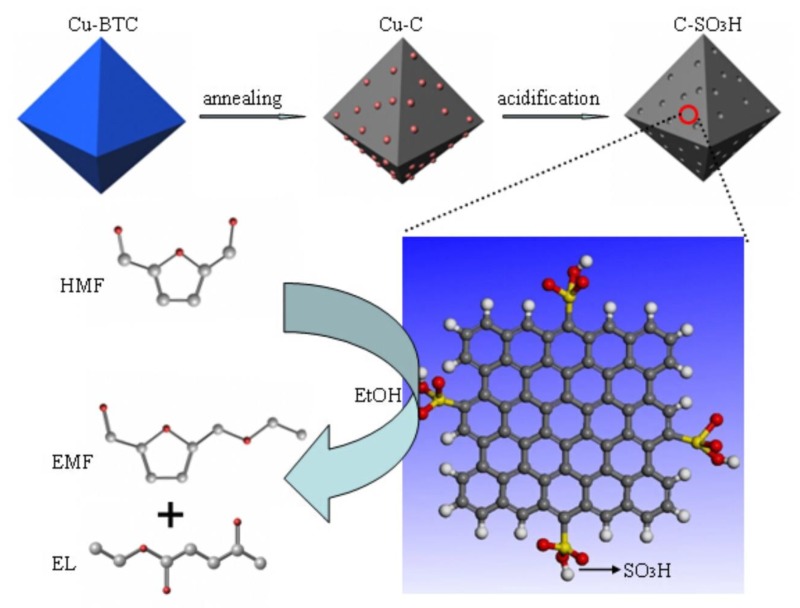
Schematic illustration of the synthesis and catalysis procedure.

The overall synthesis route to prepare sulfo-modified porous carbon nanoparticles C-SO_3_H as an efficient catalyst for conversion of HMF into EMF and EL is illustrated in Figure 1. Firstly, the unique carbon material was synthesized by a two-step strategy [24], which included the fabrication of the octahedron Cu-BTC precursor by the co-precipitation method and subsequent annealing under an N_2_ atmosphere for carbonization. The obtained composites were then reacted with H_2_SO_4_ to remove Cu as well as to introduce sulfo groups to form sulfo-doped carbon materials (C-SO_3_H). The performance of C-SO_3_H as a catalyst for the conversion of HMF into EMF and EL was tested in a micro-autoclave using ethanol as the solvent.

### 3.1. Catalyst Characterization

**Figure 2:**
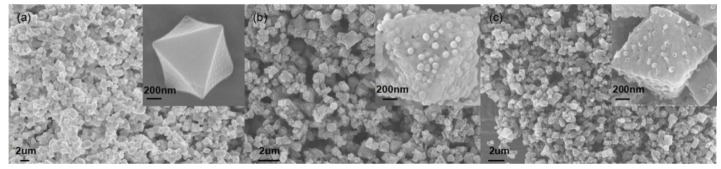
SEM images of (**a**) Cu-BTC particles, (**b**) Cu and porous carbon composites, and (**c**) C-SO_3_H particles.

**Figure 3:**
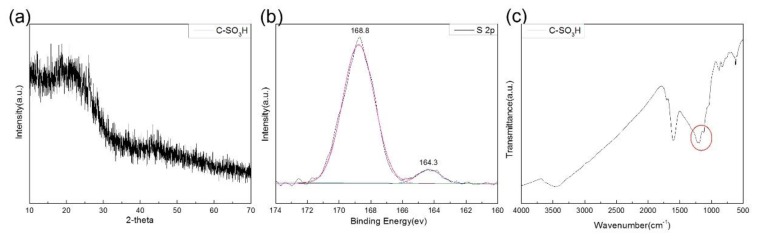
(**a**) X-ray diffraction pattern, (**b**) S2p XPS spectra, and (**c**) FT-IR spectra of C-SO_3_H.

Figure 2 shows the SEM images of the material in different stages during the preparation process. Cu-BTC particles (Figure S1a) are octahedral with a smooth surface, as shown in Figure 2a. As shown in Figure 2b, Cu and porous carbon composites derived from Cu-BTC carbonization retain an octahedral structure with Cu nanoparticle (Figure S1b) deposition on the surface of octahedral particles. The obtained Cu-C particles are then reacted with H_2_SO_4_ to remove Cu particles and generate holes on the surface (Figure 2c). The XRD pattern of the material after treatment exhibits a broad peak at approximately 2θ = 24°, which corresponds to the carbon (002) peak [24] (Figure 3a). The XPS spectra of the S2p spectrum of the material is shown in Figure 3b. The S2p peak is fitted by two peaks at the binding energies of 164.3 eV and 168.8 eV, corresponding to the S2p energy positions of thiophene sulphur and higher-oxidized sulphur forming SO_4_^2−^, respectively [25]. The absorption bands at 1116 and 1186 cm^−1^ in the FT-IR spectrum of the material are assigned to SO_3_H groups, which further confirm the successful introduction of sulfonic acid groups [26]. Moreover, there are some carboxyl and hydroxyl groups during the hot acid treatment. The results above show that the material is indeed the sulfo-modified porous carbon material (C-SO_3_H). Because the C-SO_3_H inherits the characteristic of the MOFs base, the catalyst possess large specific surface area of 377.7 m^2^/g and average pore diameter of 3.3 nm (Figure S2).

### 3.2. Catalyst Activity

The conversion of HMF into EMF and EL was carried out in a micro-autoclave utilizing heat and autogenous pressure with ethanol as reactant and reaction medium [27]. 5-ethoxymethylfurfural (EMF) and ethyl levulinate (EL) are the main reaction products observed after the reaction from GC–MS (Figure S3). A series of reaction systems with or without catalyst in different reaction conditions are compared in Table 1. The reactions carried out in the absence of a catalyst gave no product with the lower reaction temperature and low yield when the reaction temperature was high (Table 1, entries 1 and 2). However, in the same reaction conditions with C-SO_3_H as catalyst, the yield of products was remarkably increased (Table 1, entries 3 and 4). The above results indicate that C-SO_3_H is an effective catalyst for the conversion of HMF into EMF and EL. Moreover, the results of Cu-BTC and Cu-C as catalysts in the same conditions further prove the catalytic effect of C-SO_3_H (Table 1, entries 5 and 6).

**Figure 4:**
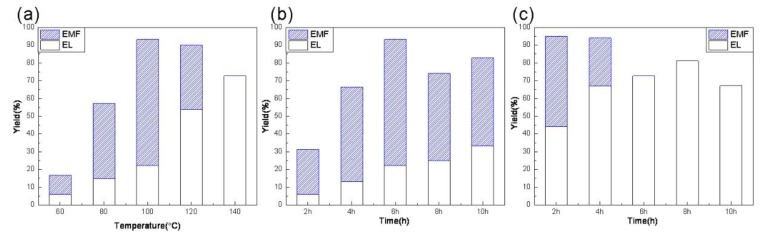
Influence of temperature and time on the reaction. (**a**) Catalytic performance of the catalyst at different temperatures for 6 h. (**b**) Catalytic performance of the catalyst at 100 °C for different lengths of time. (**c**) Catalytic performance of the catalyst at 140 °C for different lengths of time.

The effect of reaction time and temperature in the catalytic conversion of HMF into EMF and EL over the C-SO_3_H catalyst was investigated and is illustrated in Figure 4. In this reaction, the majority of the product is EMF and EL. In addition, there exist some small, unquantified by-products. Firstly, experiments were carried out for 6 h at different temperatures from 60 °C to 140 °C to study the effect of reaction temperature on this reaction (Figure 4a). A small amount of EMF was detected at a lower temperature of 60 °C, showing the lowest yield. At other temperatures, HMF had completely transformed. EMF yield increased with the increase of the reaction temperature from 60 °C to 100 °C with the maximum yield of 71%, and then declined to below 1% at 140 °C. On the other hand, a higher EL yield was obtained at a higher reaction temperature for the same reaction time and the maximum EL yield of 73% was obtained at 140 °C. The above results indicate that HMF prefers to be transformed to EL at high temperatures. The major product changes from EMF to EL with the increase of temperature.

Because the maximum yields of EMF and EL appear at 100 °C and 140 °C, respectively, different reaction times from 2 h to 10 h were carried out at 100 °C and 140 °C to probe the effect of reaction time on the yields of EMF and EL, respectively. Figure 4b shows the effect of reaction time on yield at 100 °C. No HMF was detected in this group according to GC–MS, which indicates that the conversion of HMF can be completed at 100 °C. It is obvious that EMF is the major product at 100 °C. The yield of EMF was improved with the increase of the reaction time from 2 h to 6 h, and then declined. Meanwhile, the yield of EL increased with the increase of reaction time, which clearly shows the positive effect of reaction time on the yield of EL at 100 °C.

The effect of reaction time on yield was also observed for the reactions carried out at 140 °C. Figure 4c shows that EMF yield declines from 51% to below 1% with the increase of the reaction time from 2 h to 6 h. The product was only EL when the reaction time was prolonged. Meanwhile, the yield of EL increases from 44% to a maximum of 81% when the time is prolonged from 2 h to 8 h. A lower yield of 67% was then obtained at 10 h. These results indicated that EL is the major product at the temperature of 140 °C.

**Figure 5:**
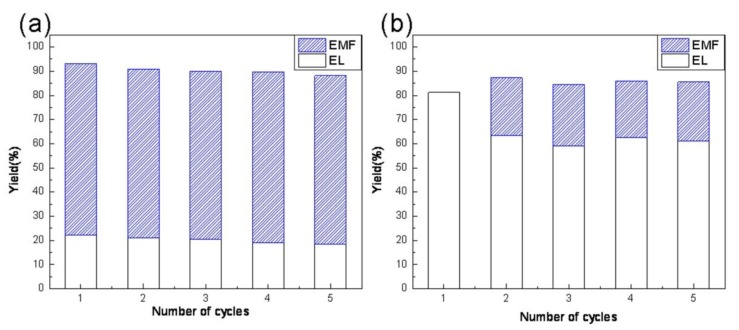
Catalyst recycling (reaction conditions: (**a**) 100 °C for 6 h; (**b**) 140 °C for 8 h).

The reuse of a catalyst is highly preferable in terms of green chemistry. The reaction conditions of 100 °C for 6 h and 140 °C for 8 h are the optimum reaction conditions to generate the maximum yields of EMF and EL, respectively. Therefore, the reusability and stability of the catalyst was investigated under the two different conditions. After the reaction, the catalyst could be easily collected by centrifugalization. The spent catalyst was then washed with ethanol and dried to remove the adsorbed products, then reused for cycling tests. The steps were repeated five times and the result is shown in Figure 5. Figure 5a reveals that the catalyst could be reused with slight loss of catalytic activity at 100 °C for 6 h. It was noted that EMF is always the major product and its yield still remains about 70% even after five cycles. The other catalytic cycles were repeated at the reaction condition for the maximum yield of EL. As shown in Figure 5b, the yield of EL declined from 81% to 64% from the first run to the second run and the yield of EMF increased from 0% to 24%. In the later cycles, there is a small decrease in the activity of the catalyst after reuse (second run: 64% yield, fifth run: 61% yield).

### 3.3. Discussion

A historical retrospect of the studies on conversion of HMF into EMF and EL reveals a common characteristic that the acid or acidic groups with high solubility and inferior reusability need to be immobilized on high-surface-area, high-active-sites, insoluble, and recyclable solid materials. The relatively high catalytic activity of C-SO_3_H is derived from its specific structure. The carbon nanoparticles derived from the pyrolysis of MOFs can keep their morphology with uniform holes and possess a large specific surface area. Due to the stability and insolubility of the carbon material, the C-SO_3_H derived from this method can be separated by centrifugation and reused. The Cu-C composites derived from pyrolysis of Cu-BTC MOFs react with H_2_SO_4_ to not only remove Cu on the surface but to also introduce the functional sulfo groups on the edge of the carbon layer [24]. Compared with other synthesis methods [22,28,29,30], this MOFs-assisted approach guarantees a carbon matrix with large specific surface area which possess more sites to introduce more acid groups, which is beneficial to the catalytic reaction. There are two reasons for choosing Cu-BTC as precursor in this work. First, Cu-BTC is commonly used and can be prepared in large quantities by a simple co-precipitation method. Moreover, Cu derived by pyrolysis of Cu-BTC can be completely removed by H_2_SO_4_. For example, the Co derived from pyrolysis of Co-BTC cannot be completely removed by H_2_SO_4_, which results in a reduction of sulfonic acid groups in the same mass of catalyst.

We notice that the results of the first and second catalysis are quite different at the condition of 140 °C for 8 h. The XRD patterns of the catalyst in the first and second catalysis show no difference (Figure S4a). However, there is a small difference in the S2p and FT-IR spectra (Figure S4b,c). The peak at the binding energy of 168.8 eV, corresponding to SO_4_^2−^, remains stable. The binding energy of the peak assigned to thiophene sulphur slightly decreases from 164.3 to 163.8 eV. A blue shift is observed for the bands assigned to SO_3_H groups in the FT-IR spectra. This indicates that the above changes exist in the catalyst after the first catalysis at high temperature. Perhaps the above factors lead to the difference in products. The specific reasons still need to be further explored in the future. 

By virtue of the specific nanostructure, the porous C-SO_3_H exhibits excellent activity for conversion of HMF into EMF and EL. It is interesting that for the catalyst C-SO_3_H, the reaction temperature and time have a great influence on the composition of the products. Different from the previous literature, different reaction temperatures and times can obtain different major products. For example, when the reaction time lasts 6 h, EMF is the absolute major product at lower reaction temperatures 60 °C, 80 °C, and 100 °C. However, EL is the major product as the reaction temperature increases to 120 °C and 140 °C. Overall, the yield of EL increases with the increase of the reaction temperature, which indicates that C-SO_3_H is preferable to catalyze the conversion of HMF into EL [31] when increasing the reaction temperature for 6 h. This confirms the report that the furan ring of HMF or EMF is prone to hydrolytic cleavage to give rise to EL at high reaction temperatures. The influence of reaction time on the product is different at different temperatures. At a lower temperature such as 100 °C, EMF is always the major product even if the further decomposition of EMF leads to an increase in EL yield when prolonging the reaction time. This indicates that the etherification of HMF to EMF is favored with respect to the degradation reaction to EL at a low temperature. As discussed before, the high selectivity to EL is clearly associated with the high temperature, which is also confirmed by the data obtained at 140 °C for different times. For a sufficient reaction time (longer than 2 h), EL is the absolute major product.

According to the previous literature, some researchers think that catalysts with only a strong Brønsted acidity such as H_2_SO_4_ react selectively to EL [11]. However, this is not absolute, because Balakrishnan found that EMF was the major product when the reaction was carried out at lower temperatures and for less time (75 °C/24 h), whereas higher temperature and longer time (120 °C/30 h) favored the formation of EL exclusively with H_2_SO_4_ as catalyst [31]. It is interesting that EMF as major product was observed with the catalysts Fe_3_O_4_@C-SO_3_H [30] and Fe_3_O_4_@SiO_2_–SH–Im–HSO_4_ even at the higher temperature of 120 °C [32]. This indicates that not all catalysts can form different major products at different reaction temperatures and times. Compared with the previous literature (Table 2), the C-SO_3_H material shows excellent activity with maximum yields of EMF and EL of 71% and 81%, respectively. Meanwhile, different from previously reported catalysts which generated only one major product, EMF or EL can be selectively obtained as the major product over C-SO_3_H by changing the reaction temperature and time. Unfortunately, the reason why EMF and EL can be selectively obtained is not clear. This can be further explored in the future.

## 4. Conclusions

In this work, a sulfonic-acid-functionalized porous carbon material (C-SO_3_H) was prepared with Cu-BTC MOFs as the precursor, showing excellent catalytic activity for the conversion of HMF into EMF and EL using ethanol as the solvent. Compared with the previous literature, a different major product (EMF or EL) can be modulated by changing the reaction temperature and time over this catalyst. The maximum yields of EMF and EL can reach 71% and 81%, respectively. This metal-free catalyst will be expected to play a more important role in industrial applications in the future.

## Figures and Tables

**Table 1:** Synthesis of EMF and EL from HMF catalyzed in different reaction conditions.

Entry	Catalyst	Reaction Condition	Yield of EMF %	Yield of EL %
1	None	100 °C/6 h	0	0
2	None	140 °C/6 h	26	4
3	C-SO_3_H	100 °C/6 h	71	22
4	C-SO_3_H	140 °C/6 h	<1	73
5	Cu-BTC	100 °C/6 h	0	0
6	Cu-C	100 °C/6 h	0	0

**Table 2:** The comparison of the catalytic performance of the present work with those reported in the literature.

Entry	Catalyst	Conversion %	Yield of EMF %	Yield of EL %
1 [11]	Z-SBA-15	100	76	23
2 [27]	NH_4_-S_5.5_	69	21	8
3 [22]	CNT-PSSA	99	-	84
4 [23]	SO_4_^2−^/ZrO_2_	100	-	30
5 [33]	K-10 clay-Al	78.9	73.2	-
6 [34]	40 wt % MCM-41-HPW	96	85	7
7 [35]	Fe_3_O4@SiO_2_-HPW	98	84	6
8 (in this work) ^a^	C-SO_3_H	100	71	22
9 (in this work) ^b^	C-SO_3_H	100	-	81

The reaction conditions: ^a^ 100 °C for 6 h; ^b^ 140 °C for 8 h.

## Supplementary Materials

The following are available online at http://www.mdpi.com/2079-4991/8/7/492/s1, Figure S1: (a) The XRD of Cu-BTC and (b) the Cu and porous carbon composites derived from Cu-BTC carbonization. Figure S2: (a) Nitrogen adsorption-desorption isotherm and (b) the corresponding pore size distribution of the C-SO_3_H. Figure S3: The general GC-MS image of the reaction products, the inset images are the MS of EL and EMF respectively. Figure S4: (a) The X-ray diffraction pattern, (b) the S2p XPS spectra and (c) the FT-IR spectra of catalysts in first and second catalysis at the condition of 140 °C for 8 h.

## References

[1] Gallezot P. (2012). Conversion of biomass to selected chemical products. Chem. Soc. Rev..

[2] Ma L.L., Wang T.J., Liu Q.Y., Zhang X.H., Ma W.C., Zhang Q.A. (2012). A review of thermal–chemical conversion of lignocellulosic biomass in China. Biotechnol. Adv..

[3] Serrano-Ruiz J.C., Luque R., Sepulveda-Escribano A. (2011). Transformations of biomass-derived platform molecules: From high added-value chemicals to fuelsvia aqueous-phase processing. Chem. Soc. Rev..

[4] Long H.L., Li X.B., Wang H., Jia J.D. (2013). Biomass resources and their bioenergy potential estimation: A review. Renew. Sustain. Energy Rev..

[5] Kocar G., Civas N. (2013). An overview of biofuels from energy crops: Current status and future prospects. Renew. Sustain. Energy Rev..

[6] Zhang J.H., Lin L., Liu S.J. (2012). Efficient production of furan derivatives from a sugar mixture by catalytic process. Energy Fuels.

[7] Román-Leshkov Y., Barrett C.J., Liu Z.Y., Dumesic J.A. (2007). Production of dimethylfuran for liquid fuels from biomass-derived carbohydrates. Nature.

[8] Deng L., Li J., Lai D.M., Fu Y., Guo Q.X. (2009). Catalytic conversion of biomass-derived carbohydrates into γ-valerolactone without using an external H_2_ supply. Angew. Chem. Int. Ed..

[9] Bond J.Q., Alonso D.M., Wang D., West R.M., Dumesic J.A. (2010). Integrated catalytic conversion of γ-valerolactone to liquid alkenes for transportation fuels. Science.

[10] Horváth I.T., Mehdi H., Fábos V., Boda L., Mika L.T. (2008). γ-Valerolactone—A sustainable liquid for energy and carbon-based chemicals. Green Chem..

[11] Lanzafame P., Temi D.M., Perathoner S., Centi G., Macario A., Aloise A., Giordano G. (2011). Etherification of 5-hydroxymethyl-2-furfural (HMF) with ethanol to biodiesel components using mesoporous solid acidic catalysts. Catal. Today.

[12] Mascal M., Nikitin E.B. (2008). Direct, high-yield conversion of cellulose into biofuel. Angew. Chem. Int. Ed..

[13] Gruter G.J.M., Dautzenberg F. (2007). Method for the Synthesis of 5-Alkoxymethylfurfural Ethers and Their Use. European Patent.

[14] Gruter G.J.M., Dautzenberg F. (2011). Method for the Synthesis of 5-Hydroxymethylfurfural Ethers and Their Use. U.S. Patent.

[15] Joshi H., Moser B.R., Toler J., Smith W.F., Terry W. (2011). Ethyl levulinate: A potential bio-based diluent for biodiesel which improves cold flow properties. Biomass Bioenergy.

[16] Bozell J.J., Moens L., Elliott D.C., Wang Y., Neuenscwander G.G., Fitzpatrick S.W., Bilski R.J., Jarnefeld J.L. (2000). Production of levulinic acid and use as a platform chemical for derived products. Resour. Conserv. Recycl..

[17] Wang Z.H., Chen Q.W. (2016). Conversion of 5-hydroxymethylfurfural into 5-ethoxymethylfurfural and ethyl levulinate catalyzed by MOF-based heteropolyacid materials. Green Chem..

[18] Dutta S., De S., Alam M.I., Abu-Omar M.M., Saha B. (2012). Direct conversion of cellulose and lignocellulosic biomass into chemicals and biofuel with metal chloride catalysts. J. Catal..

[19] Kraus G.A., Guney T. (2012). A direct synthesis of 5-alkoxymethylfurfural ethers from fructose via sulfonic acid-functionalizedionic liquids. Green Chem..

[20] Yang Y., Abu-Omar M.M., Hu C. (2012). Heteropolyacid catalyzed conversion of fructose, sucrose, and inulin to 5-ethoxymethylfurfural, a liquid biofuel candidate. Appl. Energy.

[21] Saravanamurugan S., Nguyen Van Buu O., Riisager A. (2011). Conversion of mono- and disaccharides to ethyl levulinate and ethyl pyranoside with sulfonic acid-functionalized ionic liquids. ChemSusChem.

[22] Liu R., Chen J., Huang X., Chen L., Ma L., Li X. (2013). Conversion of fructose into 5-hydroxymethylfurfural and alkyl levulinates catalyzed by sulfonic acid-functionalized carbon materials. Green Chem..

[23] Peng L., Lin L., Zhang J., Shi J., Liu S. (2011). Solid acid catalyzed glucose conversion to ethyl levulinate. Appl. Catal. A Gen..

[24] Yang Y., Zheng F.C., Xia G.L., Lun Z.Y., Chen Q.W. (2015). Experimental and theoretical investigations of nitro-group doped porous carbon as a high performance lithium-ion battery anode. J. Mater. Chem. A.

[25] Struis R., Schildhauer T., Czekaj I., Janousch M., Biollaz S., Ludwig C. (2009). Sulphur poisoning of Ni catalysts in the SNG production from biomass: A TPO/XPS/XAS study. Appl. Catal. A Gen..

[26] Zhang W., Tao H., Zhang B., Ren J., Lu G., Wang Y. (2011). One-pot synthesis of carbonaceous monolith with surface sulfonic groups and its carbonization/activation. Carbon.

[27] Lanzafame P., Barbera K., Perathoner S., Centi G., Aloise A., Migliori M., Macarib A., Nagy J.B., Giordano G. (2015). The role of acid sites induced by defects in the etherification of HMF on Silicalite-1 catalysts. J. Catal..

[28] Liu B., Zhang Z., Huang K. (2013). Cellulose sulfuric acid as a bio-supported and recyclable solid acid catalyst for the synthesis of 5-hydroxymethylfurfural and 5-ethoxymethylfurfural from fructose. Cellulose.

[29] Liu X., Xu Q., Liu J., Yin D., Su S., Ding H. (2016). Hydrolysis of cellulose into reducing sugars in ionic liquids. Fuel.

[30] Yuan Z., Zhang Z., Zheng J., Lin J. (2015). Efficient synthesis of promising liquid fuels 5-ethoxymethylfurfural from carbohydrates. Fuel.

[31] Balakrishnan M., Sacia E.R., Bell A.T. (2012). Etherification and reductive etherification of 5-(hydroxymethyl)furfural: 5-(alkoxymethyl)furfurals and 2,5-bis(alkoxymethyl)furans as potential bio-diesel candidates. Green Chem..

[32] Yin S., Sun J., Liu B., Zhang Z. (2015). Magnetic material grafted cross-linked imidazolium based polyionic liquids: An efficient acid catalyst for the synthesis of promising liquid fuel 5-ethoxymethylfurfural from carbohydrates. J. Mater. Chem. A.

[33] Liu B., Gou Z., Liu A., Zhang Z. (2015). Synthesis of furan compounds from HMF and fructose catalyzed by aluminum-exchanged K-10 clay. J. Ind. Eng. Chem..

[34] Liu A., Zhang Z., Fang Z., Liu B., Huang K. (2014). Synthesis of 5-ethoxymethylfurfural from 5-hydroxymethylfurfural and fructose in ethanol catalyzed by MCM-41 supported phosphotungstic acid. J. Ind. Eng. Chem..

[35] Wang S., Zhang Z., Liu B., Li J. (2013). Silica coated magnetic Fe_3_O_4_ nanoparticles supported phosphotungstic acid: A novel environmentally friendly catalyst for the synthesis of 5-ethoxymethylfurfural from 5-hydroxymethylfurfural and fructose. Catal. Sci. Technol..

