# Supplementary material for: Variations of Major Product Derived from Conversion of 5-Hydroxymethylfurfural over a Modified MOFs-Derived Carbon Material in Response to Reaction Conditions
Source: Nanomaterials (Basel). 2018 Jul 5;8(7):492. doi: 10.3390/nano8070492 (PMC6070794; doi:10.3390/nano8070492)
Supplement: Supplementary file 1 [file nanomaterials-08-00492-s001.pdf]

Variations of major product derived from conversion of 5-hydroxymethylfurfural over a modified MOFs-derived carbon material in response to reaction conditions

Zhenhua Wang and Qianwang Chen

Supporting Information

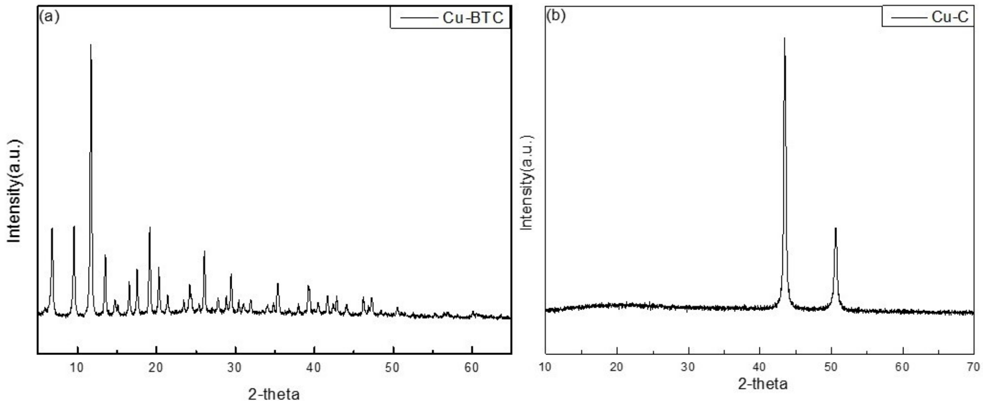

Fig. S1. (a) The XRD of Cu-BTC and (b) the Cu and porous carbon composites derived from Cu-BTC carbonization.

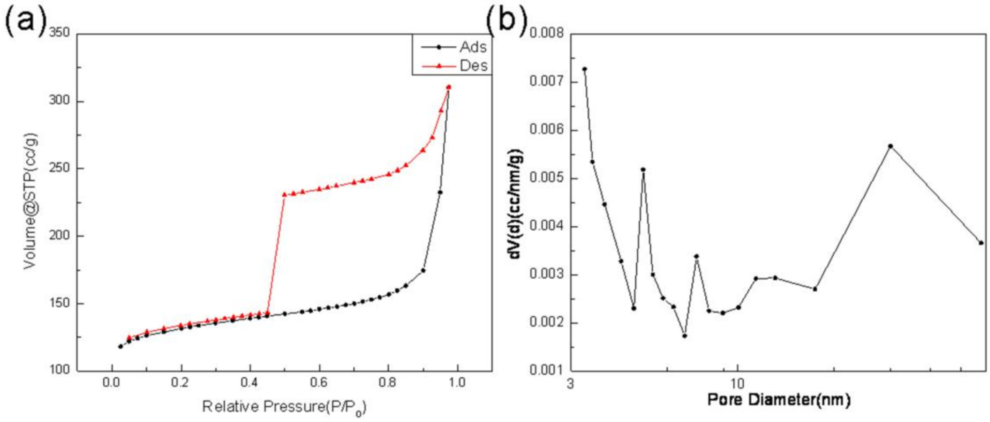

Fig. S2. (a) Nitrogen adsorption-desorption isotherm and (b) the corresponding pore size distribution of the C-SO_3H.

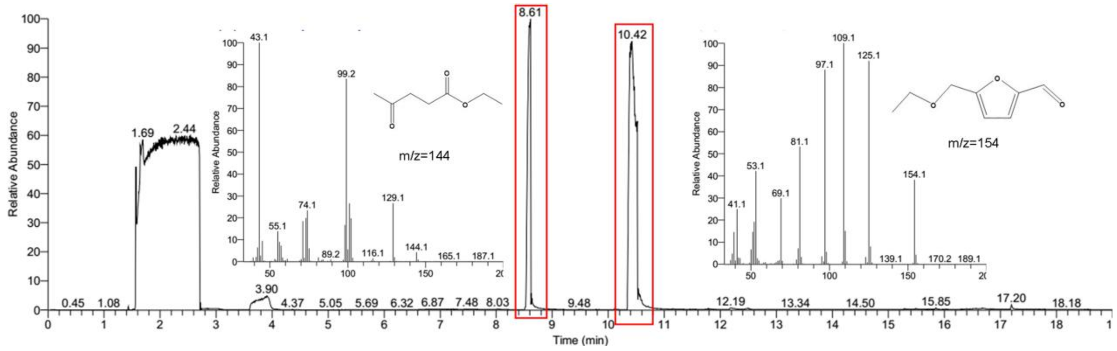

Fig. S3. The general GC-MS image of the reaction products, the inset images are the MS of EL and EMF respectively.

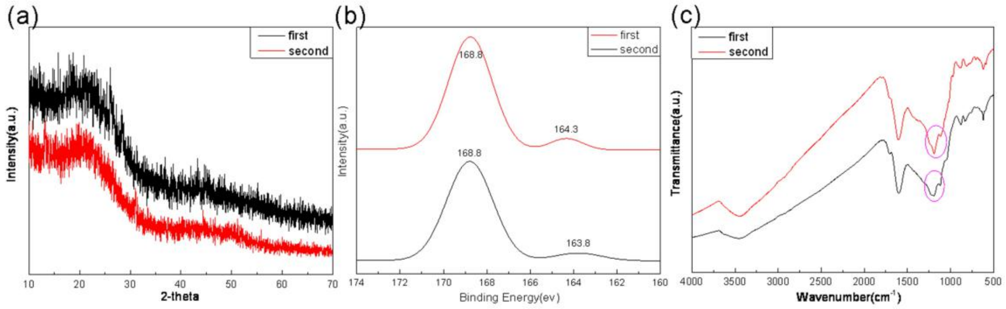

Fig. S4. (a) The X-ray diffraction pattern, (b) the S2p XPS spectra and (c) the FT-IR spectra of catalysts in first and second catalysis at the condition of 140 ºC for 8 h.
